# Comparative Study of Microwave, Pulsed Electric Fields, and High Pressure Processing on the Extraction of Antioxidants from Olive Pomace

**DOI:** 10.3390/molecules29102303

**Published:** 2024-05-14

**Authors:** Maria Tsevdou, Athina Ntzimani, Maria Katsouli, George Dimopoulos, Dimitrios Tsimogiannis, Petros Taoukis

**Affiliations:** Laboratory of Food Chemistry and Technology, School of Chemical Engineering, National Technical University of Athens, 5 Heroon Polytechniou Str., 15780 Athens, Greece; mtsevdou@chemeng.ntua.gr (M.T.); ntzimani@chemeng.ntua.gr (A.N.); mkatsouli@chemeng.ntua.gr (M.K.); gdimop@chemeng.ntua.gr (G.D.); ditsimog@chemeng.ntua.gr (D.T.)

**Keywords:** olive pomace, bioactive compounds, microwave-assisted extraction, pulsed electric fields, high-pressure processing, extraction methods

## Abstract

Olive oil production is characterized by large amounts of waste, and yet is considerably highly valued. Olive pomace can serve as a cheap source of bioactive compounds (BACs) with important antioxidant activity. Novel technologies like Pulsed Electric Fields (PEF) and High Pressure (HP) and microwave (MW) processing are considered green alternatives for the recovery of BACs. Different microwave (150–600 W), PEF (1–5 kV/cm field strength, 100–1500 pulses/15 µs width), and HP (250–650 MPa) conditions, in various product/solvent ratios, methanol concentrations, extraction temperatures, and processing times were investigated. Results indicated that the optimal MW extraction conditions were 300 W at 50 °C for 5 min using 60% *v*/*v* methanol with a product/solvent ratio of 1:10 g/mL. Similarly, the mix of 40% *v*/*v* methanol with olive pomace, treated at 650 MPa for the time needed for pressure build-up (1 min) were considered as optimal extraction conditions in the case of HP, while for PEF the optimal conditions were 60% *v*/*v* methanol with a product/solvent ratio of 1:10 g/mL, treated at 5000 pulses, followed by 1 h extraction under stirring conditions. Therefore, these alternative extraction technologies could assist the conventional practice in minimizing waste production and simultaneously align with the requirements of the circular bioeconomy concept.

## 1. Introduction

The olive oil industry is becoming one of the larger agro-food business sectors, with olive oil being one of the most widely consumed edible oils [1]. The Mediterranean countries produce roughly 98% of the world’s olive oil [2]. Greece is among the main three olive oil producers, holding the 13% of the EU production, while having the biggest EU consumption per capita (approximately 12 kg per person per year) [3]. Both the organoleptic and health-promoting qualities of olive oil have led to an increase in its consumption and production. Despite all the aforementioned, olive oil production is one of the most polluting agro-food industries, producing the commonly known olive pomace [4]. Olive pomace (OP) is the solid by-product from virgin olive oil production. It is the remaining material after removing most of the oil from the olive paste, and it consists of pieces of skin, pulp, stone, and olive kernel [5], which pose both an economic burden for manufacturers as well as a significant environmental problem.

According to Decision 2000/532/EC of the European Union Commission and to the Eurostat database classification [6], waste from agricultural activities, i.e., corn, wheat, fruit, vegetables, rice, pomace, and olive wastes are categorized as agricultural livestock waste (ALW). Massive amounts of solid waste and dark liquid effluents are produced during the industrial manufacturing process of olive oil, table olives, and olive tree cultivation [5]. Specifically, EU countries generate 9.6 million tons of waste from the olive mills each year, of which 8.4 million tons are olive pomace [6]. In 2019–2020, the world production of olive oil was characterized by huge amounts of waste (wood, branches, leaves) and by-products (olive pomace, olive mill wastewater, olive stones), counting 0.2–1.2 m^3^/t olive mill wastewater and 580–740 kg/t olive pomace of processed olives from extraction processes [2].

Although olive pomace is a by-product with reduced economic value [6], the presence and underutilization of organic compounds in the OP has created a compelling case for the development of processing strategies to add value to this by-product [7]. Olive pomace is a significant source of phenolic compounds, since only 1–2% of the total content of the phenolic compounds of olives goes into olive oil through its mechanical extraction production process (centrifugation of oil paste), while 53% and 45% remain in the liquid waste and the solid by-product (olive pomace), respectively [8]. Besides olive pomace’s current uses, including composting, soil amendment, and animal feed, it is a notable source of functional compounds and can be exploited to formulate high value-added foods. Consequently, it fosters the sustainability of the olive-oil chain which present well-recognized benefits for human health and well-being [9,10], such as anti-inflammatory, antitumor, antimicrobial, antioxidant, antidiabetic, and cardio-protective activities [11].

The recovery of antioxidant compounds from different plant sources can be achieved with extraction processes using conventional or non-conventional methods. The quality of the extracted BACs in terms of the type of compounds and their antioxidant efficiency is closely related to the characteristics of the plant source, including the geographical origin, as well as the handling and the storage conditions, but also to the involved extraction technologies [12]. Extraction is the first and the most important step to recover natural bioactive compounds (BACs) with antioxidant activity from agro-food by-products. Although the yields during conventional extractions are high, and the product obtained is of good quality, these processes require long treatment times and high temperatures, and the extracts, prior to use, must be subjected to solvent removal and purification treatments. To overcome these limitations of conventional extraction methods, new and promising extraction techniques are introduced. These techniques, such as microwave-assisted extraction [13,14], Pulsed Electric Fields (PEF) [15,16], and High Pressure (HP) processing [17,18] are referred as non-conventional extraction techniques, and have gained increasing interest due to the fact that they are amenable to automation and lead to shortened extraction times and reduced organic solvent consumption, thus mitigating pollution and reducing production costs [19].

Microwave-assisted extraction (MAE) can be classified as a green extraction technique that is mostly performed with closed vessels which operate at high pressures and temperatures and can be applied for extracting bioactive compounds from biomass [20,21,22,23]. Compared to the conventional extraction (CE) methods, MAE is known as a more environmentally friendly process with economic advantages. It is considered a novel extraction technique that can offer simplified manipulation, reduced solvent consumption, rapid and uniform heating, high thermal efficiency, lower energy input, and no pollution [24,25,26]. Among the advantages of MAE, the significantly shorter extraction time is the most important [26]. The reduction in extraction time can mainly be attributed to the difference in heating performance by the microwave technique and conventional heating. The MAE process is explained by its capacity for heating a matrix both internally and externally with no thermal gradient. Microwaves promote cell alteration and improve the recovery of the compounds of interest. Microwaves are electromagnetic fields in the frequency range from 300 MHz to 300 GHz. They are made up of two oscillating fields that are perpendicular, such as the electric field and magnetic field. The principle on which MAE is based is dielectric heating, which is the process in which a microwave electromagnetic radiation heats a dielectric material by molecular dipole rotation of the polar components present in the matrix [27,28]. The extraction mechanism of ΜAΕ is considered to involve three sequential steps described by Alupului [29]: first, separation of solutes from active sites of sample matrix under increased temperature and pressure; second, diffusion of solvent across sample matrix; and third, release of solutes from the sample matrix to the solvent. The microwave radiation immediately affects the moisture of solid material; the moisture is evaporated, generating a tremendous pressure on the cell walls which swell and rupture, leaching out the target compounds [30].

Pulsed Electric Fields (PEF) is a nonthermal food process. It is based on the exposure of cells to pulses of a high-strength electric field, which has an effect on the permeability of the cell envelope (cell membrane and cell wall). This phenomenon, commonly known as electroporation, poses a significant barrier to mass transfer to and from the cytoplasm. Processes that rely on intracellular mass transfer include dehydration, impregnation, and extraction. During the 2010s, several authors explored the effectiveness of PEF on the extraction of olive oil by pretreating the pomace or whole olives [31,32,33,34,35]. Several works also explored the scaling up of the process to a semi-industrial scale. Although PEF has been shown to be effective in increasing oil yield during the oil extraction process, little work has been carried out on the recovery of bioactive compounds from the resulting olive pomace. Nevertheless, strong evidence exists from the aforementioned studies that PEF increases the phenolic content of the resulting oil. Plant cells are especially susceptible to electroporation which highlights the benefits of using PEF for the pretreatment of plant materials prior to extraction [36]. The low energy consumption and the minimization of heat exposure make PEF pretreatment suitable for the enhancement of the extraction of thermolabile bioactive compounds from plant tissues. Several works have explored different waste-stream valorization using PEF such as spent brewer’s grains [37,38], citrus peels [15,39], sesame cake [40], potato peels [41], apple peels [42], and peach wastes [43]. Even though these materials have already been subjected to other structure-modifying treatments (e.g., comminution or pressing) during the production of the primary products, PEF was still able to improve the extraction yields of target compounds.

High Pressure (HP) technology is recognized as the most established among novel food processes, mainly applied for the inactivation of microorganisms and enzymes inducing food deterioration. Apart from that, HP has been proposed as an alternative to conventional extraction methods in order to exploit bioactive and high added-value compounds from several agricultural and food matrices of plant and animal origin [44,45,46,47,48,49], or algae [50,51,52], known as High-Pressure-assisted extraction (HPAE). The HPAE process includes mixing the raw material with the appropriate solvent, treating the mixture with HP, and then recovering the target compound from the solvent mixture. The mixture of raw material/solvent can be further concentrated, dried, or purified in order to obtain a single clear compound or fraction of compounds, which can be used either as an ingredient in the food and pharmaceutical industry or for the production of films and coatings [53]. The main process mechanism involves the disruption of the tissues, cells, and organelles enhancing mass transfer rates, increasing cell permeability and secondary metabolite diffusion, and thus leading to high extraction yields [54]. The effect of the process parameters, namely pressure level, temperature, and processing time, seems to affect differently the recovery of BACs, depending on the matrix of their source. Hence, the use of ambient or low temperature during HPAE ensures the enhanced extractability and maintenance of thermosensitive compounds. Since 2014, the FDA has recognized HPAE as an environmentally friendly extraction process due to the low energy consumption and the small amounts of volatized solvents. Additionally, the low temperature used and processing time required, as well as the high extraction yields and, in some cases, the high selectivity, are included in HPAE’s key advantages [55].

The aim of the present study was to investigate the impact of three novel extraction technologies, MAE, PEF, and HP, on the total phenolic content and the antioxidant capacity of BACs recovered from olive pomace. Samples from conventional extractions and/or untreated olive pomace were used as controls for the evaluation of the BAC’s extractability under MW, PEF, and HP conditions. The objectives of this study also include the minimization of either processing time, product mass to solvent ratio, and solvent concentration, key factors in order to ensure that alternatives to conventional extraction are more environmentally friendly.

## 2. Results and Discussion

### 2.1. Extraction of BACs from Olive Pomace with Conventional Methods

There are many solid–liquid extraction techniques, but they can be divided into two categories/methods. The first category includes the most classical techniques in which the concentration equilibrium is established between the raw material and the solvent. Practically, the raw material and the extraction solvent are mixed in a closed system, and for this reason, the system is driven to equilibrium. Techniques that involve stirring–shaking the mixture accelerate the process, but do not affect its yield; when the concentration of the extractable compounds in the solid matrix is equal to the concentration of the compounds in the liquid, then the diffusion-controlled transfer phenomena from the solid to the liquid terminate. Improvement in yields is achieved by increasing the temperature or by increasing the volume of solvent for a given mass of raw material.

The second category of solid–liquid extraction techniques includes those in which the extracted substances are removed from the plant material-solvent system (open systems). In these cases, a high concentration difference between the two phases is maintained: pure solvent is fed to the plant material, and the extract is continuously removed from the extraction system. Thanks to the high concentration difference between the two phases, no equilibrium is reached, so the driving force for mass transfer is constantly present. Theoretically, these methods can lead to exhaustive extractions. Soxhlet extraction belongs to this category. However, due to the use of increased temperatures, there are several concerns for the applicability of this classic method in the cases in which sensitive and thermolabile compounds are extracted.

Taking into consideration the above, the olive pomace was extracted conventionally according to an open system technique at room temperature using the Fixed Bed Semi-Batch Extraction (FBE) so as to achieve the complete exhaustion of BACs from the matrix, but without application of any novel assisting/accelerating technologies.

#### Recovery of BACs from Olive Pomace with Fixed Bed Semi-Batch Extraction (FBE)

The wet olive pomace was subjected to freeze drying in order to avoid any degradation of sensitive components. The dried material was further ground to powder and stored in the refrigerator. The residual moisture of the “Freeze-Dried and ground Olive Pomace” (FDOP) was determined at 2.3%. This material was used for two sequential extractions using FBE, which is with 100% methanol and then with 100% water, so as to recover into two fractions the medium and high polarity compounds, respectively.

The yield of methanolic extraction, in terms of total extracted solids (g)/100 g of dw (%), was determined to be 17.6%. Specifically, the total phenols (g GAE) per kg of the FDOP was determined to be 13.7 g GAE/kg dw. Therefore, the application of methanol for the recovery of phenolic compounds resulted in a final Dried Extract (DE) in which the TP content (TPC) amounted at 78.1 g GAE/kg DE. The specific quantification serves as a selectivity measure of the TP recovery as far as the corresponding solvent and the raw material are concerned. The sequential extraction with water yielded 3.4% total solids, 4.6 g GAE/kg dw, and the selectivity was determined to be 137 g GAE/kg DE. Methanol presents higher yields but lower selectivity than water, probably due to the ability of the specific solvent to partially recover, and also because of low polarity compounds such as lipids, chlorophylls, and waxes, which are present in FDOP. The above sequential extractions have exhausted the FDOP from phenolic compounds; therefore, we could determine the overall TPC at 18.4 g GAE/kg dw.

**Figure 1:**
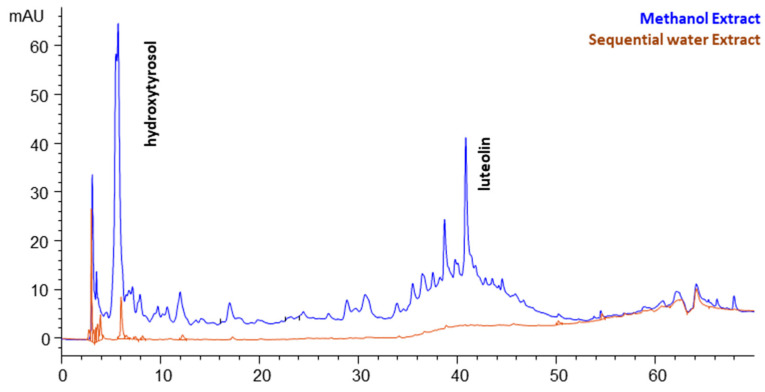
The chromatograms of the sequential methanol and water extracts recovered by the conventional fixed-bed extraction and monitored at 280 nm.

Both extracts were subjected to HPLC-DAD analyses, and Figure 1 depicts an overlay of the two chromatograms monitored at 280 nm. It can be noticed that methanol extract is a more complex mixture of components than water extract. One major compound was identified using spectral data, r.t., and finally an addition of internal standard, namely hydroxytyrosol. The second peak, with r.t = 40.8 min, presented a characteristic flavone-type UV spectrum, and it was identified as luteolin with the addition of the respective standard. In the case of water extract, hydroxytyrosol was also detected, but presented a far lower peak. Also, no new peaks are present in the water extract, just the traces of the primarily recovered compounds by methanol. Therefore, water only extracted the residual compounds of methanol extraction. According to this finding, the above reported values for water extraction could not characterize this solvent individually.

For this reason, FDOP was subjected to direct water extraction at FBE, and the results obtained show that the yields increased to 11.2% (total solids) and TPC to 9.1 g GAE/kg dw. Accordingly, the selectivity dropped to 80.9 g GAE/kg DE. We could assume that methanol and water share a fraction of compounds that are commonly recovered by both solvents. The HPLC analysis revealed that direct water extract recovered practically only hydroxytyrosol; therefore, this compound is the main common phenolic that can be recovered by both solvents.

The drying process might be critical for the stability of the BACs to be recovered. Freeze-drying is the safest drying method, but is unsustainable for drying a crude biomass at an industrial scale. For this reason, the wet olive pomace was subjected to air-drying at 40 °C in a ventilated oven for 24 h. After drying, the material was powdered and stored in a refrigerator. The residual moisture of the air-dried and ground Olive Pomace (ADOP) was determined to be 4.7%. ADOP was subsequently extracted with methanol in the FB extractor. The yield of total extracted solids was determined to be 15.3%, while the TPC at 13.5 g GAE/kg dw and the selectivity was ca. 89.7 g GAE/kg DE. The air-drying method had a minor impact on the crude extraction yield, reducing it from 17.6% to 15.3%, and also in terms of TP recovery, there was a slight decrease from 13.7 to 13.5 g GAE/kg dw. ADOP primarily extracted with methanol was further treated with water. Concerning the water extraction, the total solids yield presented a slight increase from 3.4% (FDOP) to 3.6% (ADOP), as well as the yield of TPC from 4.6 to 4.7 g GAE/kg dw. Therefore, wet olive pomace does not degrade with air-drying. On the contrary, the above evidence suggests that the microstructure of olive pomace acts as a shield against the degradation of sensitive components, and air-drying does not influence the raw material.

### 2.2. Extraction of BACs from Olive Pomace with MAE

During microwave-assisted extraction of ADOP, the extraction was carried out for 5 and 10 min at solid:liquid ratio 1:10 and 1:30 g/mL, and the extraction temperature was set at 30 and 50 °C and the microwave power at 300, 500, and 600 W. The effect of methanol-water solvent concentrations (20, 40, 60, and 100% (*v*/*v*)) was also examined. The effect of the above parameters on the extraction yield (total phenolic content, TPC) and the antioxidant activity of the extracted BACs were studied. Values of TPC and antioxidant activity of BACs extracted with MAE were compared with FBE.

#### 2.2.1. Effect of Microwave Power and Product Mass/Solvent Ratio

**Figure 2:**
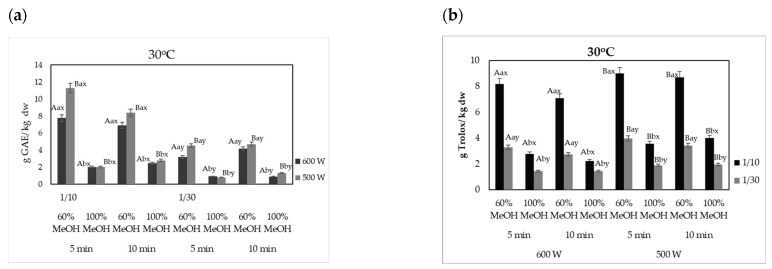
Effect of microwave power and product mass/solvent ratio on (**a**) the TPC (g GAE/kg dw); (**b**) antioxidant capacity (g Trolox/kg dw), of the microwave-assisted extracted BACs from olive pomace, at a constant temperature of 30 °C and at solid:liquid ratio 1:10 and 1:30 g/mL. Different letters in the same Figure represent significant differences (*p* < 0.05) among treatments based on Duncan’s post-hoc comparison test. Superscript letters A, B present significant differences (*p* < 0.05) between Watt; superscript letters a, b present significant differences (*p* < 0.05) between solvents; and superscript letters x, y present significant differences (*p* < 0.05) between solvent to solid ratio.

The effect of the microwave power and ADOP mass to solvent ratio on the extraction yield (expressed as g GAE/kg dw) and antioxidant activity (expressed as g Trolox/kg dw) of phenolic compounds from olive pomace is shown in Figure 2a and Figure 2b, respectively. The highest values of total phenolic content (TPC) of olive pomace extracts were recorded using 500 W at 30 °C with 60% *v*/*v* MeOH and 1:10 g/mL ADOP solid:liquid ratio, where 11.11 ± 0.13 and 9.34 ± 0.11 g GAE/kg dw extracted with for 5 and 10 min, respectively. However, when 1:30 g/mL (60% *v*/*v* MeOH) was used, TPC values were significantly lower (*p* < 0.05) and approximately 3.22 ± 0.20 and 4.19 ± 0.18 g GAE/kg dw for 5 and 10 min, respectively (Figure 2a). The aforementioned results revealed that 1:10 g/mL product solid:liquid ratio was more sufficient in recovering higher yields of BACs from olive pomace. It was observed that both TPC and antioxidant activity of the BACs recovered with MAE from olive pomace were higher (*p* < 0.05) when the microwave power was set at 500 W in contrast to 600 W, for MeOH concentration up to 60% *v*/*v*. As far as the antioxidant activity is concerned, it was similarly observed that g of Trolox per kg of dry weight were higher (*p* < 0.05) when microwave power was set at the lower values of 500 W instead of 600 W. Values of DPPH were ca. 9.09 ± 0.12 and 8.70 ± 0.16 g Trolox/kg dw when the time of extraction was 5 and 10 min and power was set at 500 W, respectively, whereas DPPH values of BACs were 8.18 ± 0.22 (5 min) g Trolox/kg dw and 7.06 ± 0.25 (10 min) g Trolox/kg dw at 600 W. Xie et al. [56] reported that the effect of microwave power on the phenolic compounds and mainly hydroxytyrosol of olive pomace increased with increasing values of power from 100 to 600 W, but the application of higher values led to a decreased effect. Results of the present study were in accordance with the respective ones of the aforementioned research, as microwave power of 500 W led to higher values of extracted BACs from olive pomace as compared to 600 W. The fundamental principle of MAE is based on heating the water within plant cells. The increased power of the microwaves accelerates the extraction of bioactive compounds due to the direct effects of microwave energy on the plant materials. Microwaves (MW) cause water molecules in the plant material to oscillate, generating heat through dielectric heating. This rapid heating helps to break down the cell walls and membranes, facilitating the release of bioactive compounds. This mechanism is effective and can reduce the extraction time compared to traditional methods. Heating with MW improves the interaction between the extracting agent and the raw material during the extraction process by loosening the cell wall matrix and the plant tissues [57]. However, excessive overheating at a very short time under the work of internal microwave radiation heating might result to chemical changes of compounds, such as phenolic, and lead to lower values of recovered BACs.

#### 2.2.2. Effect of Solvent Concentration, Temperature and Time of Extraction

**Figure 3:**
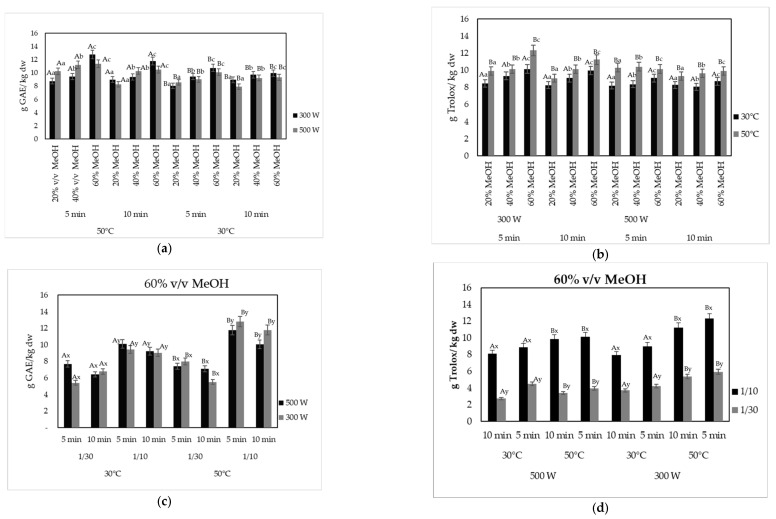
Effect of microwave power and product mass/solvent ratio on (**a**,**c**) the TPC (g GAE/kg dw); (**b**,**d**) antioxidant capacity (g Trolox/kg dw), of the microwave-assisted extracted BACs from olive pomace, using MeOH-water (*v*/*v* mixture) as solvent at 300 and 500 W for 5 and 10 min at 30 and 50 °C and at solid:liquid ratio 1:10 and 1:30 g/mL. Different letters in the same Figure represent significant differences (*p* < 0.05) among treatments based on Duncan’s post-hoc comparison test. Superscript letters A, B present significant differences (*p* < 0.05) between temperature; superscript letters a, b, and c present significant differences (*p* < 0.05) between solvents; and superscript letters x, y present significant differences (*p* < 0.05) between solvent to solid ratio.

The effect of solvent concentration, temperature, and time for the microwave-assisted extraction (MAE) of olive pomace on the recovery of the phenolic compounds and their antioxidant activity is shown in Figure 3a,c and Figure 3b,d, respectively. Results indicated that all the designing parameters, e.g., temperature, processing time, and solvent concentration, exhibited a significant impact on both the TPC and antioxidant activity values. An exception was observed only for the applied microwave power, where low watts (300 or 500 W) resulted in similar results, while higher watts (600 W) led to lower values of both TPC and antioxidant activity of the extracted BACs.

In the field of industrial processing, cutting down the extraction time while maintaining high extraction yield is a pivotal goal. As it can be observed in Figure 3a, the highest TPC values were recorded when the extraction was carried out for 5 min, followed by extraction for 10 min, showing that recovery yield was higher with decreased time of extraction and therefore showing that MAE may lead to extraction of BACs in a significantly short time. Yanık [58] reported that the extraction efficiency increased with longer irradiation times. However, irradiation for longer than 15 min had an adverse effect on extraction efficiency for both power and solvent:liquid ratio. It has been demonstrated by several authors that extraction times of longer than 10 min did not result to better results in TPC, and even a decrease after 10–15 min has been noted [59,60], suggesting that long processing times may lead to the decomposition of phenolic compounds [61]. Xie et al. [56] noted that MW extraction yields increased significantly over time before 10 min, whereas after 10 min, the concentrations of phenolic compounds tended to plateau with increasing time. The aforementioned results may be attributed to the fast heating and destruction of the biological cell structure of plant tissues under microwave conditions, providing very efficient extraction process in a shorter time [57]. The use of 20% *v*/*v* MeOH in the present study led to lower values of both TPC and DPPH, whereas 60% *v*/*v* MeOH to the highest TPC values. On the contrary, the use of 100% methanol solvent resulted in the lowest values of TPC (Figure 2a). Similar results regarding the solvent concentration were reported by Macedo et al. [62], who showed that the lowest phenolic concentration values were observed in the MAE extracts obtained with the use of 100% *v*/*v* ethanol, in contrast to 50% *v*/*v* ethanol. Solvents with lower viscosity increased the swelling of the plant materials and the contact surface between the plant matrix and the solvent, therefore enhancing the extraction yield [63]. Da Rosa et al. [61] reported that 40 and 70% ethanolic extracts showed greater antioxidant activity values than water extract obtained using MAE from olive leaves. The extract with the highest value of total phenolic content, ca. 12.80 ± 0.28 g GAE/kg dw, was obtained at 300 W using 60% *v*/*v* methanol, at 50 °C for 5 min with a product solid:liquid ratio of 1:10 g/mL. Additionally, a high TPC value (11.4 ± 0.28 g GAE/kg dw) was recorded with 1:10 g/mL, 60% *v*/*v* methanol, at 500 W and 50 °C for 5 min. In accordance with the existing literature, it was observed that higher values of both TPC and antioxidant activity of the recovered BACs were recorded when the extraction temperature was retained at 50 °C [56]. The TPC of the extracts showed a significant increase (*p* < 0.05) with temperature. High temperature (50 °C) may promote an increase in the solubility of phenolic compounds and an increase in their diffusion rate into the solvent bulk, thus increasing the mass transfer rate [56,64,65,66]. Similar results were recorded by Chanioti et al. [67].

Microwave power values of 300 and 500 W resulted in the highest TPC values with no statistically significant differences (Figure 3), while treatment at a higher power, 600 W, led to a decrease in the total phenolic content of the extracts (Figure 2). Extraction temperature is considered one of the most important factors in the field of natural components extraction, along with extraction time. The increase in temperature and pressure accelerates MAE due to the ability of extraction solvent to absorb microwave energy. High extraction temperature enhances the dissolution of phenolic substances and therefore, the mass transfer into the solvent. However, excessively high temperature can lead to the decline of the extraction efficiency due to thermal degradation of some phenolic compounds. The phenolic components, which are heat-sensitive, would be degraded due to exposure to excessive heat together and extended time [64].

Compared to conventional extraction, the green method of MAE is a high efficiency and an environmentally friendlier process. Results of the present study support the hypothesis that MAE could be a suitable alternative to conventional extraction methods achieving increased recovery of phenolic compounds even when applied for a short time [59,61]. Indeed, in MAE, the interaction between microwaves and the solvent molecules caused the temperature and internal pressure of the plant product to increase rapidly, resulting in an intense rupture of the plant cell wall, which led to a faster release of the cell compounds into the solvent and, therefore, to a higher extraction yield [61,67]. Auxiliary energy, heat generated via the electromagnetic irradiation, enables extraction processes to shorten time, reduce energy expenditure, lessen solvent usage, and thus produce higher yields comparable to traditional extraction techniques. Besides the thermal effects, MW are also reported to cause non-thermal effects that can disrupt cell structures, making the bioactive compounds more accessible for extraction [68,69]. Overall, MAE achieved similar phenolic extractability to the ones obtained by Fixed Bed Semi-Batch Extraction. Maximum recovery yields of BACs from ADOP with MAE and Fixed Bed Semi-Batch Extraction were ca. 12.8 and 18.2 g GAE/kg dw, respectively. The aforementioned yield was recovered in a significantly shorter (*p* < 0.05) extraction time with MW, 5 min instead of 120 min (2 h), respectively. Similar results were noted for the antioxidant activity of the recovered BACs, 12.3 ± 0.29 vs. 12.7 ± 0.18 g Trolox/kg dw, with MAE and Fixed Bed Semi-Batch Extraction, respectively, confirming that MAE led to similar phenolic contents with similar antioxidant activity in shorter times as compared to Fixed Bed Semi-Batch Extraction.

### 2.3. Extraction of BACS from Olive Pomace Assisted with PEF

Results for the PEF-assisted extraction at different solid to liquid ratios, solvent methanol contents, PEF treatment conditions, and extraction times are presented in Figure 3, as g of GAE/kg extracted material (in dry basis, i.e., correcting for the material’s initial moisture content). In all treatments studied, the extraction time had a significant effect on the yield of total phenolic compounds extracted from olive pomace. For samples not treated with PEF, at time zero (immediately after solvent addition), the total phenolic content of the extract was equal to 1.8 g GAE/kg dry material for a solid to liquid ratio of 1:10, irrespective of the type of solvent used. This value corresponds to the phenolic compounds that are immediately released into the solvent during the “washing” step of the extraction and was gradually increased with extraction time, reaching 8.2 g GAE/kg dry material for samples extracted with pure water and 15.7 g GAE/kg dry material for 60% *v*/*v* methanol, respectively. Thus, the efficiency of the extraction at long extraction times was strongly dependent on the solvent type.

**Figure 4:**
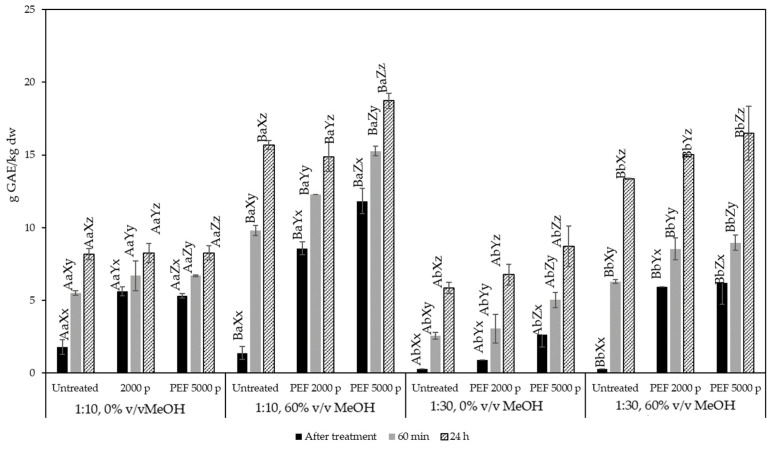
Total phenolic content of olive pomace extracts expressed as g GAE/kg dry olive pomace for untreated and PEF-treated samples (2000 and 5000 pulses) at a solid:liquid ratio of 1:10 and 1:30 g/mL. Different letters represent significant differences (*p* < 0.05) among treatments based on Duncan’s post-hoc comparison test. Superscript letters A, B present significant differences (*p* < 0.05) between solvents; superscript letters a, b present significant differences (*p* < 0.05) between solvent to solid ratio; superscript letters X, Y, and Z present significant differences (*p* < 0.05) between untreated and PEF treated; and superscript letters x, y, and z present significant differences (*p* < 0.05) between process times.

PEF treatments applied had a significant effect on the extraction of total phenolic compounds. Figure 4 demonstrates that PEF had a significant effect on the extracted phenolic compounds immediately after processing. However, this effect was attributed to the significant processing time (approximately 5 to 9 min) for PEF-treated samples. This was a deliberate processing choice. So as to avoid the heating of the sample, the pulse delivery frequency was kept relatively low (5–10 Hz). When the TPC yield of samples treated with PEF were compared to those from untreated samples extracted at the same times, it was observed that the apparent yield increase immediately after processing was attributed to time alone and was independent of PEF processing.

PEF treatment did have a significant effect on the extraction yield of phenolic compounds after 1 h and after 24 h of extraction. The most remarkable results were observed for a solid to liquid ratio equal to 1:10 g/mL at 1 h and a solvent containing 60% *v*/*v* MeOH. A treatment of 5000 pulses was able to increase the extraction yield from 9.8 g GAE/kg for the untreated sample up to 15.3 g GAE/kg at 1 h of extraction, while at 24 h of extraction the same treatment was able to increase the yield up to 18.6 g GAE/kg, compared to the untreated sample which achieved 15.7 g GAE/kg. Similar effects were observed at a solid to liquid ratio equal to 1:30 g/mL.

**Figure 5:**
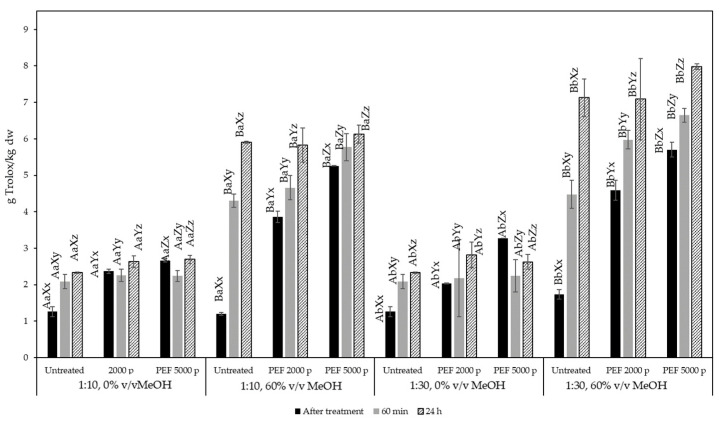
Total antioxidant capacity of olive pomace extracts expressed as g Trolox equivalents/kg dry olive pomace for untreated and PEF-treated samples (2000 and 5000 pulses) at a solid:liquid ratio of 1:10 and 1:30 g/mL. Different letters represent significant differences (*p* < 0.05) among treatments based on Duncan’s post-hoc comparison test. Superscript letters A, B present significant differences (*p* < 0.05) between solvents; superscript letters a, b present significant differences (*p* < 0.05) between solvent to solid ratio; superscript letters X, Y, and Z present significant differences (*p* < 0.05) between untreated and PEF treated; and superscript letters x, y, and z present significant differences (*p* < 0.05) between process times.

Figure 5 shows the results of the recovered antioxidant capacity, expressed in g Trolox equivalents/kg dw, obtained from various extraction conditions of olive pomace. It can be observed that when pure water was used as a solvent, neither treatment intensity nor extraction time contributed to an increase in the extracted antioxidant capacity in all solid:liquid ratios tested. This is contrary to the observations made for the TPC, suggesting that compounds extracted in the water may qualify as phenolic compounds but do not exhibit strong antioxidant capacity. On the other hand, when 60% *v*/*v* methanol was used as a solvent, the total antioxidant capacity of the extracts was correlated with the total phenolic content.

Compared to PEF treatment of other plant tissues, the effects observed on the extraction of phenolic compounds from olive pomace may at first appear unspectacular. Contrary to the treatment of other plant tissues, the improvement of extraction of phenolic compounds from olive pomace poses several challenges. The material itself exhibits several barriers that may hinder the extraction, such as residual oil content, the presence of ligneous fragments, and the presence of waxy olive skin particles. Bousetta et al. [70] reported that the required field strength for effective treatment of flaxseed hulls exceeded 10 kV/cm and remarked that materials with a high ligninocellulosic content and low moisture may require a treatment intensity upwards of 20 kV/cm and a specific energy input upwards of 300 kJ/kg. In our case, the field strength of 4.5 was imposed by a technical limitation of the equipment, for the specific food matrix. It is possible that at more intense electric field conditions, the extraction yield would be significantly higher. However, this is unlikely because the TPC yields obtained with PEF treatment at 24 h of extraction approach the theoretically maximum content as measured in the exhaustive extraction step. Roselló-Soto et al. [71] treated olive pomace with PEF and High Voltage Electric Discharge (HVED) and obtained better outcomes at specific energy inputs exceeding 100 kJ/kg and better extraction outcomes for HVED, which is a more intense electrical treatment. On the contrary, other plant-based residues that are more homogeneous and susceptible to electroporation may be treated at milder conditions. Frontuto et al. [41] studied the effect of PEF pretreatment on the extraction kinetics of phenolic compounds from potato peels at a maximum energy input of 5 kJ/kg and found that the extraction could be shortened by 96 min. Similarly, Peiró et al. [15] were able to increase the extractability of phenolic compounds from lemon residues by 300% at a field strength of 7 kV/cm and a specific energy input of 7.6 kJ/kg.

### 2.4. Extraction of BACS from Olive Pomace with HPAE

During high-pressure-assisted extraction (HPAE) of milled olive pomace, the extraction was carried out for the time needed for pressure build-up at the desirable level, i.e., 40–60 s to 20 min, using different product solid:liquid ratios and methanol concentrations at room temperature. The effect of the above parameters on the extraction yield (TPC) and the antioxidant activity of BACs was evaluated. Values of TPC and antioxidant activity of BACs extracted with HPAE were compared with that of HP-untreated samples that remained for the corresponding processing time in calm.

#### 2.4.1. Effect of Processing Parameters on the Total Phenolic Content of Extracted BACs

**Figure 6:**
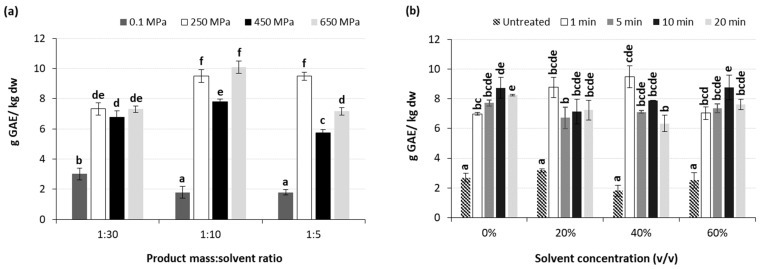
Effect of (**a**) applied pressure and dried mass to solvent ratio at a methanol concentration of 40% *v*/*v*, and (**b**) processing time and solvent concentration (*v*/*v*) at a dried mass to solvent ratio of 1:10 g/mL and pressure level of 250 MPa, on the TPC (g GAE/kg dw) of the high-pressure-assisted extracted BACs from olive pomace at ambient temperature. Different letters in the same Figure represent significant differences (*p* < 0.05) among treatments based on Duncan’s post-hoc comparison test.

Figure 6a shows the effect of the applied pressure level and the product mass to methanol ratio used on the TPC of high pressure extracted BACs. Results indicate that an increase in the applied pressure from atmospheric to high pressure levels led to significant (*p* < 0.001) increase in the TPC of the extracted BACs. Increasing pressure levels improved the solvent’s efficacy, boosting both the density and solubility of polar compounds. Additionally, the increased pressure facilitated deeper solvent penetration into cells by disrupting cell walls, thereby augmenting permeability and consequently enhancing the mass-transfer rate [18]. However, a further increase in the applied pressure from 250 to 650 MPa did not only significantly affect, but in some cases led to, a decrease in the TPC of the extracted BACs. A similar trend has been previously reported by Cascaes Teles et al. [49] during the HPAE of phenolic compounds from grape pomace at the pressure range of 50–200 MPa, where the application of 100 MPa led to lower levels of TPC compared to that after treatment at either 50 or 200 MPa.

Regarding the effect of product mass to solvent ratio, results indicate that this ratio of 1:10 g/mL exhibited the highest TPC for HP extracted BACs for all applied high pressures. On the other hand, a product mass to solvent ratio of 1:30 g/mL was found to be more effective in the case of BACs extraction under atmospheric pressure. In this case, this can be attributed to the combined effects of increased contact surface area, improved solvent penetration, enhanced mass transfer, and reduced diffusion resistance, contributing to the observed increase in extractability of phenolic compounds from olive pomace with higher solid to liquid ratios. In particular, as the solid to liquid ratio decreases, more solvent comes into contact with the olive pomace, enhancing the interaction between the solvent and the phenolic compounds present in the pomace and facilitating their extraction into the liquid phase. Additionally, a lower solid to liquid ratio is related to higher availability of solvent to penetrate into the pores and cell walls of the olive pomace particles and access to the phenolic compounds trapped within the cellular structure of the pomace, thus leading to higher extraction yields. Last but not least, when decreasing the solid to liquid ratio, the reduction in the diffusion resistance encountered by the solvent molecules as they move through the pomace matrix is promoted. This reduction in diffusion resistance allows for faster and more thorough extraction of phenolic compounds, leading to higher extraction yields [72]. On the other hand, when BACs extraction is performed under HP conditions, the effect of applied pressure on the plant cell membranes is so intense that the need for high amounts of solvent is reduced. The highest TPC was achieved for the samples treated at 650 MPa using 40% *v*/*v* methanol at a solid to liquid ratio of 1:10 g/mL, followed by the samples treated at 250 MPa using the same methanol concentration at a solid to liquid ratio of either 1:10 or 1:5 g/mL, with slight yet not statistically significant differences (Figure 6a).

Several studies have shown that the application of HPAE in plant tissues improves the extractability of phenolic compounds, both in terms of yield and processing time, due to the instant and uniform transfer of the high pressures to the tissue, resulting in a rapid achievement of an equilibrium during HPAE [73]. In our study, processing time under HP conditions did not affect the TPC of the extracted BACs, with the exception of samples mixed with 40% *v*/*v* MeOH, for which a significant (*p* < 0.05) decrease in the TPC was observed when the processing time was increased (Figure 6b). This is in agreement with previous studies, where an increase in the processing time when treated at 200 MPa led to lower levels of TPC after the first 5 min of extraction of phenolic compounds from grape pomace mixed with sodium acetate buffer using a solid to liquid ratio of 1:8 [50]. Similarly, Chen et al. [74] reported an increase in TPC during the first minutes of the HP processing of red wine, followed by a decrease and stabilization in the TPC values between 5 and 60 min of compression. This was attributed to the breakdown of phenolic compounds in low molecular weight compounds, which cannot be quantified with the Folin–Ciocalteu assay.

**Figure 7:**
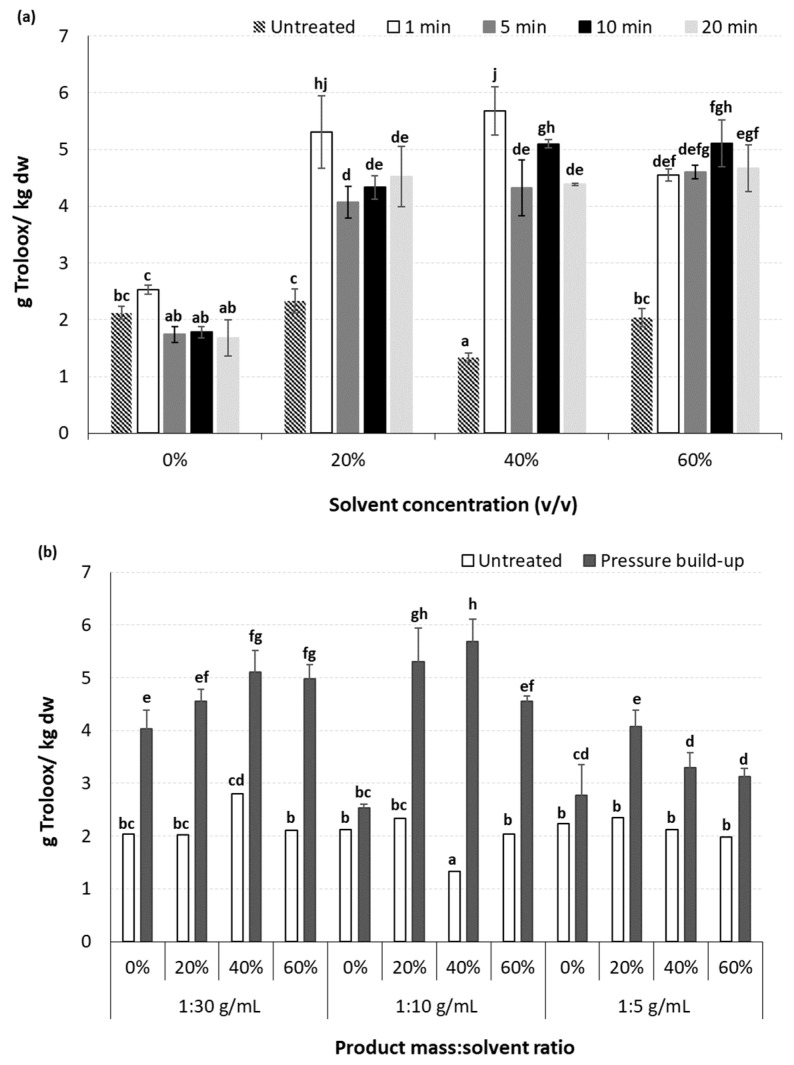
Effect of (**a**) processing time and methanol concentration at a constant solid:liquid ratio of 1:10 g/mL and applied pressure of 250 MPa, and (**b**) applied pressure and product solid:liquid ratio on the antioxidant capacity (g Trolox/kg dw) of the high-pressure-assisted extracted BACs from olive pomace, at ambient temperature. Different letters in the same Figure represent significant differences (*p* < 0.05) among treatments based on Duncan’s post-hoc comparison test.

It is worth noting that when applying low pressures (i.e., 250 MPa) and using water as a solvent, the TPC of extracted BACs significantly (*p* < 0.05) depended on the processing time, mainly during the first 10 min of extraction (Figure 7b). In particular, TPC values exhibited a percent increase of approximately 230% after 10 min under HPAE versus that of 160% after pressure build-up time, compared to the untreated samples. Several studies have shown that during HPAE, either applied as a pre-treatment or not, the processing time affects the extractability of BACs from plant origin matrices. This is attributed to the fact that: (i) longer processing times allow for more thorough penetration of the solvent into the matrix; (ii) prolonged exposure to high pressures can further disrupt the cell walls and membranes, thus promoting the release of phenolic compounds trapped within the cellular matrix; (iii) there is more time for the solvent to interact with BACs present, allowing for greater solubilization of BACs; and (iv) some BACs exhibits time-dependent release kinetics, requiring sufficient processing time for optimal extraction [18,47]. Moreover, after 10 min of compression at 250 MPa using water as a solvent, the TPC value of extracted BACs was similar to those obtained when using higher methanol concentrations of either 20% or 40% *v*/*v*. This observation is of high significance in extraction applications where the use of “green” solvents is required, with the benefit of achieving similar or even higher TPC in significant shorter processing times than those of conventional extractions methodologies.

#### 2.4.2. Effect of Processing Parameters on the Antioxidant Activity of Extracted BACs

The antioxidant activity of the extracted BACs was determined through the DPPH assay and was expressed as Trolox equivalent antioxidant capacity (Figure 7). The use of aqueous solutions of methanol instead of pure deionized water had a significant (*p* < 0.05) effect on the antioxidant activity of the HP-extracted BACs, presenting in most cases more than a 100% increase in the obtained Trolox equivalent values (Figure 7a). However, an increase in the methanol concentration from 20 to 60% *v*/*v* did not affect the antioxidant activity of the extracted BACs. Regarding the effect of HPAE processing time, this did not seem to enhance the antioxidant activity of the extracted BACs, with the exception of the case where pure deionized water was used as a solvent, for which significant (*p* < 0.05) decrease in the values of Trolox equivalent was observed (Figure 7a). As in the case of PEF-assisted extraction, this observation is in contrast to the results obtained for TPC. This can be related to the fact that either during HPAE, phenolic compounds with slight or no antioxidant capacity are also extracted, or under certain processing conditions (e.g., pressure levels, variations on processing temperature, etc.) they may collectively contribute to higher extraction yields of phenolic compounds during HPAE, potentially leading to pro-oxidant behavior instead of antioxidant activity [75].

The increase in solvent concentration from pure deionized water to methanol aqueous solutions resulted in a significant (*p* < 0.05) increase in the antioxidant capacity of the extracted BACs (Figure 7b). However, a further increase in methanol concentration from 20 to 60% *v*/*v* did not lead to further enhancement of the antioxidant capacity of the extracted phenolic compounds, which is in accordance with the results from the TPC, indicating that the majority of the extracted compounds presents the expected antioxidant activity. Regarding the product mass to solvent ratios tested, it was observed that higher ratios are more efficient in the enhancement of the extractability and antioxidant capacity of the extracted BACs (statistical mean values of Trolox equivalent: 4.98 ± 0.13, 4.55 ± 0.10 and 3.13 ± 0.30 g/kg dw for samples treated with ratios of 1:30, 1:10 and 1:5 g/mL, respectively). The results of this study revealed that the product mass to solvent ratios of 1:30 and 1:10 g/mL exhibited the best results on antioxidant capacity of the extracted BACs. For both these ratios, an increase in the applied pressure resulted in significant (*p* < 0.05) improvement of the antioxidant activity of extracted BACs only in the case where 60% *v*/*v* MeOH was used. Again, and in agreement with the results obtained for TPC, when pure water was used as a solvent, an increase in the applied pressure of up to 450 MPa led to higher values of Trolox equivalent for the extracted phenolic compounds (Figure 7b).

For a novel technology to be characterized as efficient for BACs extraction yield, solvent consumption and processing time, are considered. Overall, the results from HPAE of phenolic compounds from olive pomace indicated that the recommended HP conditions for the most efficient BACs extraction are the use of 40% *v*/*v* MeOH with a product to mass ratio of 1:10 g/mL, treated at 650 MPa for the time needed for pressure build-up (ca. 1 min). In these conditions, the TPC of extracted BACs and their antioxidant capacity were increased by 460% and 330%, respectively, compared to untreated samples.

### 2.5. Analyses of Extracts at Optimal Extraction Conditions

As presented above, the main parameters examined for the optimization of extraction conditions were the total solid as well as the total phenolic yields. These indices served well for the needs of the numerous extraction experiments carried out so as to determine the optimal conditions of each technique. At the same time, a series of HPLC-DAD analyses on selected samples provided chromatograms similar to those presented in Figure 1 concerning FBE, and verified the uniformity of peak patterns. Practically, the same components were recovered in all assisted extractions, as expected, since similar mixtures of methanol and water were used. The relative concentrations of individual components varied between the different extraction protocols.

The final extracts, corresponding to the optimal conditions, were analyzed using LC-MS. A characteristic set of the identified components is presented in Table 1, and corresponds to the optimal extract of PEF pretreatment. It should be noted that the same peaks were identified in all optimal samples of the studied assisted extractions. All of the compounds have been previously reported in the literature as components of olive products and by-products [76,77,78,79,80,81].

However, the compounds of high interest and abundance in the extracts were hydroxytyrosol and luteolin. These compounds, as well as Total Phenols (TP) and total flavonoids, were quantified, and the results are presented in Table 2. From the comparison of the individual technologies, it can be observed that optimal PEF extraction resulted in the highest yield of TP (15.7 g GAE/kg dw) and a quite high selectivity, as determined with the TP per dried extract (DE), but produced poor results concerning the compounds of high interest. Maybe the harsh conditions of 5000 electric pulses lead to the degradation of delicate phenolic compounds such as hydroxytyrosol and flavonoids. The optimal MW extraction presented lower performance as far as the yield of TP is concerned (11.4 g GAE/kg dw), but superior results regarding the concentration of bioactives per dried extract. It can be observed that this is the method of highest selectivity concerning both total and individual phenols. Indeed, the optimal MW extraction exhibited the lowest total solids yield (8.6%), while optimal HPAE and PEF amounted 11.3 and 14.5%, respectively. This means that the conditions of optimal MW extraction prevent inert materials to be efficiently extracted, and therefore produce more concentrated extracts as far as the phenolic compounds are concerned.

## 3. Materials and Methods

### 3.1. Materials

Fresh olive pomace was sourced from a local olive processing plant carrying out two-phase olive oil extraction from olives of the Manaki variety cultivated in the Peloponnese region. The raw pomace had a moisture content of 45% *w*/*w* on a wet basis and a residual oil content of 7.4% *w*/*w* on a dry basis. The pomace was kept at 0 °C until further processing. For experiments where dry material was required, the pomace was either air-dried at 40 °C for 24 h or freeze-dried at −52 °C and 0.080 mbar for 48 h using a Christ Alpha 1–4 LD plus freeze-dryer (Martin Christ Gefriertrocknungsanlagen GmbH, Harz, Germany). The dry pomace was stored in sealed polyethylene-polypropylene sachets at room temperature.

### 3.2. Chemicals and Reagents

Folin–Ciocalteu’s reagent, citric acid, gallic acid, 2,2-diphenyl-1-picrylhydrazyl radical (DPPH), methanol (HPLC grade), ethanol, water (HPLC grade), acetonitrile (HPLC grade), sodium carbonate, sodium sulfate, sodium citrate, and sodium acetate were purchased from Sigma Aldrich Chemical Co. (St. Louis, MO, USA). Phenolic standards: hydroxytyrosol, caffeic acid, vanillin, rutin, and luteolin were procured from Sigma-Aldrich (St. Louis, MO, USA), and oleuropein was provided from Extrasynthese (Genay, France).

### 3.3. Fixed Bed Semi-Batch Extraction of BACs

The fixed-bed semi-batch extraction has been described by Kaloudi et al. [82], and actually belongs to the technique of percolation. In percolation, the material is packed in a column (fixed bed) without any shaking or stirring. The system is fed with pure solvent through the inlet of the extractor, which passes through the pores of the material, extracts the components, and exits the system from the outlet as an extract. For the needs of the current experiments, the extractor was filled each time with 20 g of dried and grinded olive pomace, the solvent was passed through the material at a flow of 3 mL/min using a peristaltic pump (Millipore, Burlington, MA, USA) at room temperature. The exiting extracts initially had very high concentration of the extractable components, and presented dark color, which, however, decreased with time and tended asymptotically to zero, and then the produced extract was almost totally discolorized. The duration of extractions ranged between 90–120 min until obtaining a practically discolorized extract from the outlet of the extractor. The solvents used for the fixed-bed extractions were methanol and water, applied successively in two-stage extractions. The extractions were run in duplicate experiments.

### 3.4. Microwave-Assisted-Extraction (MAE) of BACs

MAE was carried out using a laboratory scale apparatus (Nanjing Xianou Instruments Manufacture Co., Ltd., Nanjing, China) using different mixtures of methanol:water (20:80, 40:60, and 60:40 *v*/*v*) as solvent, at 300–500–600 W for 5, 10, and 30 min at different solid/liquid ratios (1:10 g/mL and 1:30 g/mL). The samples of the olive pomace used for MAE were air-dried at 40 °C for 24 h and then grinded. Supernatants were analyzed for total phenolic content, antioxidant activity, and extract dry weight using the protocols described below. Table 3 displays the experimental extraction conditions that were used. Temperature and microwave radiation were continuously monitored throughout the procedure. The extractions were run in duplicate experiments.

### 3.5. PEF-Assisted-Extraction of BACs

Pulsed electric field treatment was carried out in a 60 mL chamber with stainless steel plates at an electrode distance of 4 cm. Pretreatments were carried out by suspending fresh olive pomace in sufficient solvent volume to yield final methanol concentrations of 0 (water only) and 60% *v*/*v* methanol at solid to liquid ratios of 1:10 and 1:30 g/mL, calculated in terms of pomace dry weight. In order to achieve comparable treatments between the different solvent systems and solid-to-liquid ratios, the electrical conductivity of the samples was adjusted to 800 µs/cm using a 2 M NaCl solution.

PEF treatments were carried out within the solvent by delivering bipolar, nearly rectangular pulses of 15 µs width at 4.5 kV/cm electric field strength. The pulses were delivered to the suspensions at a frequency of 5–10 Hz to avoid significant heating of the sample. Pulses were generated using a pilot scale PEF system capable of processing small batch volumes of sample (ELCRACK HVP-5 PEF system, DIL, Quackenbrück, Germany). Treatments were carried out at 2000 (70 kJ/kg specific energy input) and 5000 pulses (175 kJ/kg specific energy input). Following PEF treatment, samples were placed on a platform shaker rack where extraction was continued under agitation (180 rpm). Samples were withdrawn from the suspensions at predetermined time intervals and were centrifuged at 10,000× *g* for 5 min. Supernatants were analyzed for total phenolic content, antioxidant activity, and extract dry weight using the protocols described below. The extractions were run in duplicate experiments.

### 3.6. HP-Assisted-Extraction (HPAE) of BACs

**Figure 8:**
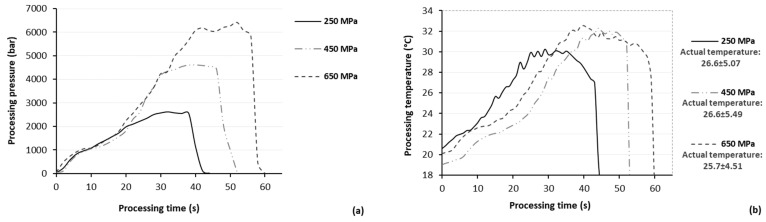
Continuous monitoring of (**a**) pressure and (**b**) temperature during HPAE of bioactive compounds from olive pomace, presenting the pressure build-up and the adiabatic heating during the performance of the studied processing conditions.

An appropriate amount of grinded air-dried (40 °C for 24 h) olive pomace was transferred into multilayer (PE-aluminum-PET12) pouches and diluted with methanol at a ratio of 1:5, 1:10, and 1:30 g/mL solid to liquid exactly prior to HP treatment. The efficacy of HP on the extractability of BACs from olive pomace was investigated at different HP conditions (pressure range: 250–650 MPa; temperature: 25 °C, and processing time: 40 s–20 min, where 40 s indicate the time required to pressure build-up). HPAE was conducted in a 1.5 L pilot-scale HP unit (Food Pressure Unit FPU 1.01, Resato International BV, Assen, The Netherlands), which operates in the pressure and temperature range of 100–1000 MPa and 0–90 °C, respectively, using water as the transmitting fluid. During HP treatment, continuous monitoring of both pressure (Figure 8a) and temperature (Figure 8b) was performed, using FPU 1.01 software (Resato International BV, Assen, The Netherlands) and PC400 version 2.1 software (Campbell Scientific, Logan, UT, USA), respectively.

After HPAE, samples were centrifuged at 3000× *g* for 5 min. The supernatant was filtered using 0.45 μm syringe filter, collected, and further analyzed for total phenolic content, antioxidant activity, and extract dry weight using the protocols described below.

HP-untreated samples were also prepared using the same mass to solvent ratios as above, and remained without any stirring for processing time equal to a fully conducted HP-cycle, i.e., sample introduction into the equipment/compression/decompression/sample extraction from the equipment, in order to serve as control samples. All experiments were performed in duplicate.

### 3.7. Total Phenolic Content (TPC)

The Folin–Ciocalteu method, as described by Chanioti and Tzia [83], was used to determine the total phenolic content, with gallic acid serving as standard. In total, 7.9 mL of deionized water, 0.5 mL of the Folin–Ciocalteu reagent, and 0.1 mL of extract were added to the mixture and vortexed. After that, 1.5 mL of saturated Na_2_CO_3_ was added, the mixture was vortexed again, and then incubated for 2 h in darkness. The solution’s absorbance was then determined at 765 nm using a spectrophotometer (Hitachi, Tokyo, Japan, U-2900 UV/Vis, 200 V). The results were given as g of gallic acid equivalents (GAE)/kg of dry weight of olive pomace (dw) (g GAE/kg dw).

### 3.8. Antioxidant Activity of BACs

Antioxidant activity was determined using the DPPH assay, as described by Chanioti and Tzia [83]. In total, 0.1 mL of the extract and 3.9 mL of DPPH radical solution (0.0025 g/100 mL methanol) were mixed, and after 20 min remaining in darkness, the mixture was measured at 515 nm using a spectrophotometer (Hitachi, U-2900 UV/Vis, 200 V). A Trolox calibration curve in the range 0.04–0.28 mg/mL was prepared (R^2^ = 0.998), and data were expressed in terms of Trolox equivalent antioxidant capacity (g Trolox equivalents/kg dry weight of olive pomace (dw)).

### 3.9. HPLC-DAD Analyses of the Extracts

In order to identify and quantify the main components of the extracts, a series of HPLC-DAD analyses were performed. The HPLC system consisted of a quaternary pump (HP 1100 gradient pump), a degasser (HP 1100), an autosampler (Agilent Infinity 1260), and a Diode Array Detector (Hewlett Packard, Waldbronn, Germany). A ZORBAX Eclipse XDB-C18 column (5 μm, 250 × 4.6 mm, Agilent, Santa Clara, CA, USA) was used at room temperature, while the samples were injected after filtration (0.45 μm, PVDF syringe filters, Teknokroma, Barcelona, Spain). The gradient method, including three solvents (water, methanol, acetonitrile, acidified with TFA 0.2% *v*/*v*), has been extensively described in previous papers [81,84,85]. The detection was performed at 230, 280, and 360 nm, and the elaboration of chromatographic data was performed on a ChemStation for LC 3D software version B.04.06 (Agilent Technologies, Santa Clara, CA, USA). Hydroxytyrosol (Extrasynthese, France) and luteolin (Extrasynthese, France) standards were used for the development of the respective calibration curves for the quantification of the compounds in extracts.

### 3.10. LC-ESI/MS Analyses

The LC-ESI/MS system consisted in an Agilent 1200 chromatograph (Santa Clara, CA, USA), equipped with an Eclipse XDB-C18, 3.5 μm, 2.1 × 150 mm column (Agilent Technologies, Santa Clara, CA, USA), and coupled with a Sciex 3500 Triple Quad mass spectrometer (Sciex, Framingham, MA, USA). The mobile phase contained two solvents coded A and B. Solvent A consisted of 2% MeOH, 0.1% HCOOH, 97.9% 5 mM HCOONH_4_ in water, and Solvent B 0.1% HCOOH, 99.9% 5 mM HCOONH_4_ in MeOH. The composition of the mobile phase changed with linear gradients of Solvents A and B, according to the program presented in Table 4.

The SCIEX QTRAP 3500 LC-MS system was operated in negative electrospray ionization mode. The experiment was performed with “Q3 MS (Q3)” scan type, with Q3 at unit resolution and a scan rate of 200 Da/s between 80 and 600 Da; curtain gas was set at 20 psi, ion source temperature at 550 °C, and ion source gas GS1 and GS2 at 45 and 50 psi, respectively. The ionspray voltage was set at −4500 V, declustering potential at −60 V, entrance potential at −10 and collision exit potential at −9 V.

### 3.11. Statistical Analysis

The experimental data were analyzed using analysis of variance (ANOVA) (STATISTICA 7, Statsoft Inc., Tulsa, OK, USA), with significant differences in mean values estimated at the probability threshold *p* < 0.05. Duncan’s multiple range test was performed to separate the data’s means when significant differences were found.

## 4. Conclusions

This work explored the extraction of antioxidants from olive pomace using various methods, including the conventional technique of FBE, and novel extraction processes of microwaves, and nonthermal PEF and HP. For MAE, the optimal conditions involved a product mass to solvent ratio of 1:10 g/mL, use of 60% *v*/*v* methanol as the extraction solvent, and a microwave power of 300 W at 50 °C for 5 min. Regarding PEF, the application of 5000 pulses followed by 1 h of extraction using a product to mass ratio of 1:10 g/mL with 60% *v*/*v* methanol were assessed as the optimal extraction conditions. In the case of HPAE, the recommended conditions for efficient BACs extraction were the use of 40% *v*/*v* methanol as a solvent, a solid to liquid ratio of 1:10 g/mL, and treatment at 650 MPa for the time needed for pressure build-up (approximately 1 min). Comparative evaluation of the proposed alternative extraction technologies favored PEF over MAE- and HP-assisted extraction, regarding the recovered total phenols, as it led to almost 85% recovery of total phenols versus 70 and 55% for MAE and HP, respectively, compared to FBE. However, concerning the antioxidant activity, MAE presented superior results, exhibiting 97% of the antioxidant activity obtained from FBE, in contrast to PEF and HP, where only 45% of the antioxidant activity obtained from conventional treated samples was gained.

The findings underscore the potential of these alternative technologies in the valorization of olive oil side streams in adherence to the bio-circularity philosophy. With regard to applicability and scalability of the three proposed technologies, it should be pointed out that all have been adopted in commercial food processing. Microwave (MW) and high pressure (HP) processing are more widely applied in the food industry followed by pulsed electric fields (PEF), and for all technologies, industrial equipment is available and adaptable to specific process applications. MW and PEF technologies require lower capital costs than HP. On the other hand, MW shows certain limitations due to the induced increase in the food temperature. In terms of processing time, the results showed that during HPAE, the lowest processing times were noted. The environmental impact of all three proposed technologies is comparable and would allow us to classify them as green [53]. Each of the proposed technologies has its advantages and disadvantages, in terms of their industrial application and in terms of their effect on food, hence benchmarking will depend on processing and final product requirements. Nonetheless, further research and analysis is warranted in order to confirm the feasibility and quantitatively compare the economic viability and environmental impact of the proposed alternative processes versus conventional extraction.

## Figures and Tables

**Table 1:** Tentative identification of phenolic compounds and their derivatives in olive pomace by HPLC-ESI/MS.

Peak	Tentative Identification	Rt (min)	*m*/*z*
1	Quinic acid	6.54	191.1
2	Hydroxytyrosol	18.87	153.1
3	Hydroxytyrosol glucoside	18.87	315.2
4	Protocatechic acid	18.87	153.1
5	Hydroxytyrosol oxidized	18.87	151.1
6	Oleoside derivative	21.3	407.2
7	Hydroxylated/Acidic derivative of decarboxymethyl elenolic acid	22.02	199.1
8	Tyrosol Glucoside or Hydroxybenzoic acid glucoside	22.50	299.2
9	Tyrosol	23.69	137.1
10	Oleoside/secologanoside or isomer	23.88	389.2
11	Caffeic acid	25.17	179.1
12	7-Epiloganin	25.18	389.2
13	Oleuropein aglycone derivative	27.25	377
14	Coumaric acid	27.80	163.1
15	Oleuropein	28.75	539.3
16	Luteolin-7-*O*-rutinoside	28.99	593.3
17	Elenolic acid	29.65	241.1
18	Oleocanthalic acid	30.08	319.2
19	Hydroxytyrosol acetate	30.09	195.1
20	p-coumaroyl-6-oleoside	31.48	535.3
21	Luteolin-4-*O*-glucoside/Luteolin-3-*O*-glucoside	31.67	447.2
22	Comselogoside (p-coumaroyl-6-secologanoside)	32.22	535.3
23	Ligstroside	32.49	523.3
24	Luteolin	33.44	285.2
25	6-*O*-[(2E)-2,6-dimethyl-8-hydroxy-2-octenoyloxy] secologanoside	34.01	557.3
26	Apigenin	34.82	269.1

**Table 2:** The quantifications of the key-bioactive compounds from olive pomace, extracted conventionally and by the three novel assisting technologies at optimal conditions.

	Total Phenols C (g GAE/kg) per		Hydroxytyrosol C (g/kg) per		Luteolin C (g/kg) per		Total Flavonoids C (g/kg) per	
	dw	DE	dw	DE	dw	DE	dw	DE
**FBE** 100% MeOH/1:15/25 °C/60 min	13.7	78.1	3.5	16.0	0.6	2.7	0.8	3.9
**MW** 60% MeOH/1:10/300 W/50 °C/5 min	11.4	132	2.7	31.5	0.4	4.3	0.7	7.5
**PEF** 60% MeOH/1:10/5000 pulses/25 °C/60 min stirring	15.7	108.2	2.0	13.6	0.2	1.3	0.3	2.4
**HPAE** 40% MeOH/1:10/650 MPa/25 °C/1 min	10.1	89.8	0.9	7.8	>0.1	0.2	0.1	0.6

dw: dry weight of olive pomace, DE: dried extract.

**Table 3:** Experimental MAE conditions for olive pomace by-product.

Microwave Frequency (Watt)	Temperature (°C)	Process Time (min)	Solvent (MeOH) Concentration (% *v*/*v*)	Product Mass/ Solvent (g/mL)
300 500 600	30 50	5 10 30	20 40 60 100	1:10 1:30

**Table 4:** The flow and evolution of mobile phase with linear gradients during LC-ESI/MS analyses.

t (min)	Flow (μL/min)	Solvent A (%)	Solvent B (%)
0	100	97	3
1.5	100	97	3
28	100	0	100
43	100	0	100
43.1	150	97	3
58	150	97	3
60	100	97	3

## Data Availability

The original contributions presented in the study are included in the article, further inquiries can be directed to the corresponding author.
